# Bis(3-acetyl­pyridine-κ*N*)diaqua­bis­(seleno­cyanato-κ*N*)cobalt(II)

**DOI:** 10.1107/S1600536812018387

**Published:** 2012-04-28

**Authors:** Julia Werner, Jan Boeckmann, Inke Jess, Christian Näther

**Affiliations:** a Institut für Anorganische Chemie, Christian-Albrechts-Universität Kiel, Max-Eyth-Strasse 2, 24118 Kiel, Germany

## Abstract

In the crystal structure of the title compound, [Co(NCSe)_2_(C_7_H_7_NO)_2_(H_2_O)_2_], the Co^2+^ cation is coordinated by two seleno­cyanate anions, two 3-acetyl­pyridine ligands and two water mol­ecules within a slightly distorted CoN_4_O_2_ octa­hedron. The asymmetric unit consists of one Co^2+^ cation, which is located on a center of inversion, as well as one seleno­cyanate anion, one 3-acetyl­pyridine ligand and one water mol­ecule in general positions. Whereas one of the water H atoms makes a classical O—H⋯O hydrogen bond, the other shows a O—H⋯Se inter­action.

## Related literature

For general background to this work, see: Näther & Greve (2003 ▶). For the synthesis, structures and properties of the corresponding compounds with pyridine, see: Boeckmann & Näther (2010 ▶, 2011 ▶, 2012 ▶).
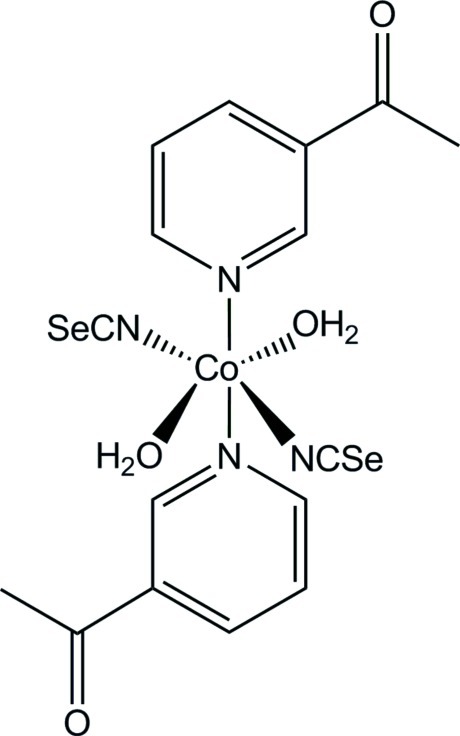


## Experimental

### 

#### Crystal data

[Co(NCSe)_2_(C_7_H_7_NO)_2_(H_2_O)_2_]
*M* *_r_* = 547.19
Monoclinic, 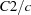

*a* = 19.1098 (6) Å
*b* = 9.0064 (4) Å
*c* = 14.9734 (5) Å
β = 128.203 (2)°
*V* = 2025.13 (13) Å^3^
*Z* = 4
Mo *K*α radiation
μ = 4.47 mm^−1^
*T* = 293 K
0.35 × 0.27 × 0.19 mm

#### Data collection

Stoe IPDS-2 diffractometer
Absorption correction: numerical (*X-SHAPE* and *X-RED32*; Stoe & Cie, 2008 ▶) *T*_min_ = 0.240, *T*_max_ = 0.423
16689 measured reflections
2409 independent reflections
2260 reflections with *I* > 2σ(*I*)
*R*_int_ = 0.028

#### Refinement

*R*[*F* ^2^ > 2σ(*F* ^2^)] = 0.029
*wR*(*F* ^2^) = 0.066
*S* = 1.12
2409 reflections
125 parameters
H-atom parameters constrained
Δρ_max_ = 0.50 e Å^−3^
Δρ_min_ = −0.45 e Å^−3^

### 

Data collection: *X-AREA* (Stoe & Cie, 2008 ▶); cell refinement: *X-AREA*; data reduction: *X-AREA*; program(s) used to solve structure: *SHELXS97* (Sheldrick, 2008 ▶); program(s) used to refine structure: *SHELXL97* (Sheldrick, 2008 ▶); molecular graphics: *XP* in *SHELXTL* (Sheldrick, 2008 ▶) and *DIAMOND* (Brandenburg, 2011 ▶).; software used to prepare material for publication: *publCIF* (Westrip, 2010) ▶.

## Supplementary Material

Crystal structure: contains datablock(s) I, global. DOI: 10.1107/S1600536812018387/bt5898sup1.cif

Structure factors: contains datablock(s) I. DOI: 10.1107/S1600536812018387/bt5898Isup2.hkl

Additional supplementary materials:  crystallographic information; 3D view; checkCIF report

## Figures and Tables

**Table 1:** Hydrogen-bond geometry (Å, °)

*D*—H⋯*A*	*D*—H	H⋯*A*	*D*⋯*A*	*D*—H⋯*A*
O1—H1*O*⋯O11^i^	0.82	1.97	2.780 (2)	169
O1—H2*O*⋯Se1^ii^	0.82	2.57	3.338 (2)	157

Symmetry codes: (i) 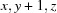; (ii) 
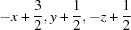
.

**Fig. 1.**
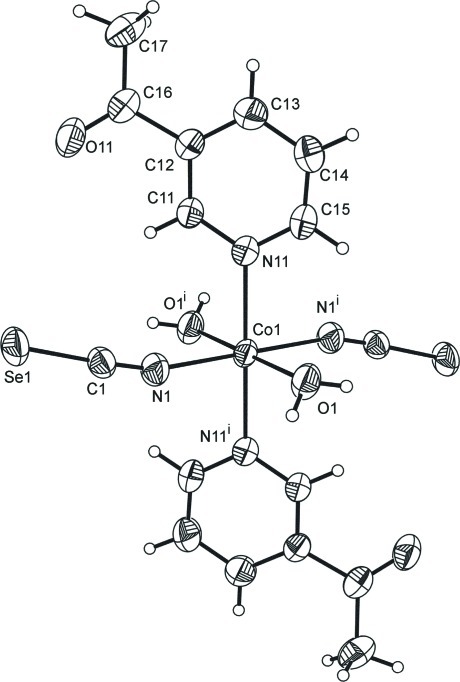
: Crystal structure of the title compound with labelling and displacement ellipsoids drawn at the 50% probability level. Symmetry code: i = -x+1,-y+1,-z.

**Fig. 2.**
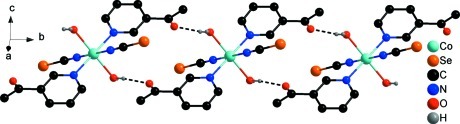
: Crystal structure showing the discrete complexes that are linked by hydrogen bonding into chains that elongate in the direction of the crystallographic b axis. Hydrogen bonding is shown as dashed lines and for clarification only the O-H H atoms are shown.

## supplementary crystallographic information

### Comment

Recently we have reported on the magnetic properties of 1D coordination polymers based on paramagnetic transition metal cations that are coordinated by pyridine as co-ligand and that are µ-1,3-bridged into chains by thio- or selenocyanato anions (Näther & Greve, 2003). Dependent on the nature of the metal cation, anti- or ferromagnetic ordering is observed and for the compounds with Co(II) as cation we have found a slow relaxation of the magnetization (Boeckmann & Näther 2010, 2011 and 2012). To investigate the influence of the co-ligand on the magnetic properties we tried to prepare similar compounds based on 3-acetylpyridine, which resulted in the formation of the title compound in which the anionic ligands are only terminal N-coordinated. Further investigations also show that this compound cannot be transformed into the corresponding 1D coordination polymers and therefore, it was characterized only by single crystal X-ray diffraction.

In the crystal structure the cobalt(II) cations are coordinated by four nitrogen atoms of two terminal *N*-bonded selenocyanato anions and two terminal bonded 3-acetylpyridine co-ligands as well as two water molecules into discrete complexes (Fig. 1). The coordination polyhedron of the Co cations can be described as a slightly distorted octahedron with the Co cation located on a centre of inversion. The discrete cobalt complexes are bridged by two pairs of intermolecular O—H···O hydrogen bonding into 16-membered rings that are located on centres of inversion (Fig. 2). These rings are further linked into hydrogen bonded chains that are parallel to the *b* axis (Fig. 2 and Table 1).

### Experimental

Potassium selenocyanate and 3-acetylpyridine were purchased from Alfa Aesar and the Co(NO_3_)_2_.6 H_2_O obtained from Merck. The title compound was prepared by the reaction of 72.8 mg Co(NO_3_)_2_.6 H_2_O (0.25 mmol), 64.8 mg KSeCN (0.45 mmol) and 54.6 µ*L* 3-acetylpyridine (0.50 mmol) in 1.5 mL H_2_O at RT in a closed 3 ml snap cap vial. After three days pink blocks of the title compound were obtained.

### Refinement

The C-H H atoms were positioned with idealized geometry (methyl H atoms allowed to rotate but not to tip) and were refined isotropic with *U*_iso_(H) = 1.2 *U*_eq_(C) for aromatic H atoms (1.5 for methyl H atoms) using a riding model with C—H = 0.93 Å (aromatic) and with C—H = 0.96 Å (methyl). The O-H H atom were located in difference map, their bond lengths set to ideal values of 0.84 Å and afterwards they were refined using a riding model with *U*_iso_(H) = 1.5 *U*_eq_(O).

### Crystal data

**Table:**

[Co(NCSe)_2_(C_7_H_7_NO)_2_(H_2_O)_2_]	*F*(000) = 1076
*M**_r_* = 547.19	*D*_x_ = 1.795 Mg m^−^^3^
Monoclinic, *C*2/*c*	Mo *K*α radiation, λ = 0.71073 Å
Hall symbol: -C 2yc	Cell parameters from 16689 reflections
*a* = 19.1098 (6) Å	θ = 2.6–27.9°
*b* = 9.0064 (4) Å	µ = 4.47 mm^−^^1^
*c* = 14.9734 (5) Å	*T* = 293 K
β = 128.203 (2)°	Block, pink
*V* = 2025.13 (13) Å^3^	0.35 × 0.27 × 0.19 mm
*Z* = 4	

### Data collection

**Table:**

Stoe IPDS-2 diffractometer	2409 independent reflections
Radiation source: fine-focus sealed tube	2260 reflections with *I* > 2σ(*I*)
Graphite monochromator	*R*_int_ = 0.028
ω scans	θ_max_ = 27.9°, θ_min_ = 2.6°
Absorption correction: numerical (*X-SHAPE* and *X-RED32*; Stoe & Cie, 2008)	*h* = −25→24
*T*_min_ = 0.240, *T*_max_ = 0.423	*k* = −11→11
16689 measured reflections	*l* = −19→19

### Refinement

**Table:**

Refinement on *F*^2^	Primary atom site location: structure-invariant direct methods
Least-squares matrix: full	Secondary atom site location: difference Fourier map
*R*[*F*^2^ > 2σ(*F*^2^)] = 0.029	Hydrogen site location: inferred from neighbouring sites
*wR*(*F*^2^) = 0.066	H-atom parameters constrained
*S* = 1.12	*w* = 1/[σ^2^(*F*_o_^2^) + (0.0275*P*)^2^ + 2.5784*P*] where *P* = (*F*_o_^2^ + 2*F*_c_^2^)/3
2409 reflections	(Δ/σ)_max_ < 0.001
125 parameters	Δρ_max_ = 0.50 e Å^−^^3^
0 restraints	Δρ_min_ = −0.45 e Å^−^^3^

### Special details

**Table:**

Geometry. All esds (except the esd in the dihedral angle between two l.s. planes) are estimated using the full covariance matrix. The cell esds are taken into account individually in the estimation of esds in distances, angles and torsion angles; correlations between esds in cell parameters are only used when they are defined by crystal symmetry. An approximate (isotropic) treatment of cell esds is used for estimating esds involving l.s. planes.
Refinement. Refinement of F^2^ against ALL reflections. The weighted R-factor wR and goodness of fit S are based on F^2^, conventional R-factors R are based on F, with F set to zero for negative F^2^. The threshold expression of F^2^ > 2sigma(F^2^) is used only for calculating R-factors(gt) etc. and is not relevant to the choice of reflections for refinement. R-factors based on F^2^ are statistically about twice as large as those based on F, and R- factors based on ALL data will be even larger.

### Fractional atomic coordinates and isotropic or equivalent isotropic displacement parameters (Å^2^)

**Table:**

	*x*	*y*	*z*	*U*_iso_*/*U*_eq_	
Co1	0.5000	0.5000	0.0000	0.03364 (10)	
Se1	0.725951 (16)	0.09800 (3)	0.20771 (2)	0.05020 (9)	
C1	0.65776 (13)	0.2600 (2)	0.13699 (18)	0.0372 (4)	
N1	0.61139 (12)	0.3598 (2)	0.09124 (17)	0.0431 (4)	
O1	0.55451 (11)	0.64233 (17)	0.14168 (13)	0.0450 (4)	
H1O	0.5265	0.7200	0.1249	0.068*	
H2O	0.6080	0.6587	0.1792	0.068*	
N11	0.43968 (12)	0.36819 (19)	0.05628 (16)	0.0393 (4)	
C11	0.44407 (14)	0.2201 (2)	0.06235 (18)	0.0373 (4)	
H11	0.4741	0.1709	0.0406	0.045*	
C12	0.40582 (14)	0.1366 (2)	0.09965 (17)	0.0370 (4)	
C13	0.36145 (18)	0.2091 (3)	0.1322 (2)	0.0499 (6)	
H13	0.3339	0.1558	0.1560	0.060*	
C14	0.3585 (2)	0.3627 (3)	0.1289 (3)	0.0574 (7)	
H14	0.3303	0.4146	0.1524	0.069*	
C15	0.39775 (18)	0.4364 (3)	0.0905 (2)	0.0497 (6)	
H15	0.3952	0.5395	0.0880	0.060*	
C16	0.41692 (16)	−0.0284 (2)	0.10701 (19)	0.0423 (5)	
C17	0.3525 (2)	−0.1217 (3)	0.1065 (3)	0.0614 (7)	
H17A	0.2945	−0.1129	0.0342	0.092*	
H17B	0.3500	−0.0890	0.1655	0.092*	
H17C	0.3713	−0.2236	0.1194	0.092*	
O11	0.47857 (13)	−0.08133 (19)	0.11398 (17)	0.0558 (5)	

### Atomic displacement parameters (Å^2^)

**Table:**

	*U*^11^	*U*^22^	*U*^33^	*U*^12^	*U*^13^	*U*^23^
Co1	0.03083 (19)	0.02384 (18)	0.0412 (2)	0.00276 (13)	0.01973 (16)	0.00073 (14)
Se1	0.04556 (14)	0.04665 (15)	0.06007 (16)	0.01964 (10)	0.03351 (12)	0.01574 (11)
C1	0.0309 (9)	0.0380 (10)	0.0403 (11)	0.0027 (8)	0.0208 (9)	−0.0010 (8)
N1	0.0335 (9)	0.0369 (9)	0.0504 (10)	0.0059 (7)	0.0217 (8)	0.0035 (8)
O1	0.0406 (8)	0.0337 (8)	0.0491 (9)	0.0013 (6)	0.0218 (7)	−0.0061 (6)
N11	0.0400 (9)	0.0303 (9)	0.0499 (10)	0.0021 (7)	0.0289 (8)	0.0011 (7)
C11	0.0387 (10)	0.0302 (10)	0.0435 (11)	0.0021 (8)	0.0258 (9)	−0.0007 (8)
C12	0.0389 (10)	0.0306 (10)	0.0382 (10)	0.0001 (8)	0.0222 (9)	0.0004 (8)
C13	0.0598 (14)	0.0450 (13)	0.0603 (14)	−0.0013 (11)	0.0449 (13)	−0.0002 (11)
C14	0.0723 (18)	0.0462 (14)	0.0800 (18)	0.0040 (13)	0.0603 (16)	−0.0056 (13)
C15	0.0586 (15)	0.0316 (11)	0.0690 (16)	0.0030 (10)	0.0445 (14)	−0.0046 (10)
C16	0.0500 (12)	0.0330 (10)	0.0390 (10)	0.0001 (9)	0.0250 (10)	0.0013 (8)
C17	0.0768 (19)	0.0403 (13)	0.0705 (18)	−0.0095 (13)	0.0473 (16)	0.0032 (12)
O11	0.0598 (11)	0.0342 (8)	0.0701 (12)	0.0088 (8)	0.0386 (10)	0.0034 (8)

### Geometric parameters (Å, º)

**Table:**

Co1—N1^i^	2.0962 (18)	C11—H11	0.9300
Co1—N1	2.0962 (18)	C12—C13	1.376 (3)
Co1—O1^i^	2.1202 (15)	C12—C16	1.495 (3)
Co1—O1	2.1202 (15)	C13—C14	1.384 (4)
Co1—N11	2.1562 (19)	C13—H13	0.9300
Co1—N11^i^	2.1562 (19)	C14—C15	1.367 (4)
Se1—C1	1.800 (2)	C14—H14	0.9300
C1—N1	1.146 (3)	C15—H15	0.9300
O1—H1O	0.8201	C16—O11	1.214 (3)
O1—H2O	0.8200	C16—C17	1.487 (4)
N11—C11	1.336 (3)	C17—H17A	0.9600
N11—C15	1.338 (3)	C17—H17B	0.9600
C11—C12	1.384 (3)	C17—H17C	0.9600
			
N1^i^—Co1—N1	180.00 (8)	N11—C11—H11	118.6
N1^i^—Co1—O1^i^	92.26 (7)	C12—C11—H11	118.6
N1—Co1—O1^i^	87.74 (7)	C13—C12—C11	118.7 (2)
N1^i^—Co1—O1	87.74 (7)	C13—C12—C16	122.4 (2)
N1—Co1—O1	92.26 (7)	C11—C12—C16	118.9 (2)
O1^i^—Co1—O1	180.00 (9)	C12—C13—C14	118.9 (2)
N1^i^—Co1—N11	90.77 (7)	C12—C13—H13	120.6
N1—Co1—N11	89.23 (7)	C14—C13—H13	120.6
O1^i^—Co1—N11	90.43 (7)	C15—C14—C13	118.6 (2)
O1—Co1—N11	89.57 (7)	C15—C14—H14	120.7
N1^i^—Co1—N11^i^	89.23 (7)	C13—C14—H14	120.7
N1—Co1—N11^i^	90.77 (7)	N11—C15—C14	123.6 (2)
O1^i^—Co1—N11^i^	89.57 (7)	N11—C15—H15	118.2
O1—Co1—N11^i^	90.43 (7)	C14—C15—H15	118.2
N11—Co1—N11^i^	180.00 (9)	O11—C16—C17	122.3 (2)
N1—C1—Se1	177.1 (2)	O11—C16—C12	118.8 (2)
C1—N1—Co1	163.86 (18)	C17—C16—C12	118.8 (2)
Co1—O1—H1O	112.7	C16—C17—H17A	109.5
Co1—O1—H2O	115.2	C16—C17—H17B	109.5
H1O—O1—H2O	110.9	H17A—C17—H17B	109.5
C11—N11—C15	117.4 (2)	C16—C17—H17C	109.5
C11—N11—Co1	123.37 (15)	H17A—C17—H17C	109.5
C15—N11—Co1	119.25 (15)	H17B—C17—H17C	109.5
N11—C11—C12	122.9 (2)		

Symmetry code: (i) −*x*+1, −*y*+1, −*z*.

### Hydrogen-bond geometry (Å, º)

**Table:**

*D*—H···*A*	*D*—H	H···*A*	*D*···*A*	*D*—H···*A*
O1—H1*O*···O11^ii^	0.82	1.97	2.780 (2)	169
O1—H2*O*···Se1^iii^	0.82	2.57	3.338 (2)	157

Symmetry codes: (ii) *x*, *y*+1, *z*; (iii) −*x*+3/2, *y*+1/2, −*z*+1/2.

## References

[bb1] Boeckmann, J. & Näther, C. (2010). *Dalton Trans.* **39**, 11019–11026. 10.1039/c0dt00904k 20949144

[bb2] Boeckmann, J. & Näther, C. (2011). *Chem. Commun.* **47**, 7104–7106. 10.1039/c1cc12273h 21617809

[bb3] Boeckmann, J. & Näther, C. (2012). *Polyhedron*, **31**, 587–595.

[bb4] Brandenburg, K. (2011). *DIAMOND* Crystal Impact GbR, Bonn, Germany.

[bb5] Näther, C. & Greve, J. (2003). *J. Solid State Chem.* **176**, 259–265.

[bb6] Sheldrick, G. M. (2008). *Acta Cryst.* A**64**, 112–122. 10.1107/S0108767307043930 18156677

[bb7] Stoe & Cie (2008). *X-AREA*, *X-RED32* and *X-SHAPE* Stoe & Cie, Darmstadt, Germany.

[bb8] Westrip, S. P. (2010). *J. Appl. Cryst.* **43**, 920–925.

